# Preparation of high Fischer ratio peptides from seabuckthorn (*Hippophae rhamnoides* L.) seed meal, characterization and functional evaluation

**DOI:** 10.1016/j.fochx.2025.102523

**Published:** 2025-05-05

**Authors:** Yuanju Zheng, Yunxi Zhou, Yichao Pei, Zhuoling Feng, Michael Yuen, Hywel Yuen, Qiang Peng

**Affiliations:** a College of Food Science and Engineering, Northwest A&F University, Yangling, China; b Puredia Limited, Xining, China

**Keywords:** Enzyme hydrolysis, Branched-chain amino acid, Aromatic amino acid, Silicon forecasts

## Abstract

In order to study the changes before and after the preparation of high F-ratio peptides, seabuckthorn seed meal high F-ratio peptides (HFP) were prepared by using alkaline protease and papain with activated charcoal adsorption, which resulted in an F-ratio of 21.626 at a peptide yield of 22.54 ± 0.86 %. A comparison of seabuckthorn seed meal peptides (HP) and their high F-ratio counterparts revealed alterations in the secondary structure of the peptides, a decline in fluorescence intensity. Additionally, a shift in the microstructure towards a more loosely packed configuration was observed. Sensory evaluation yielded no significant differences. The physicochemical properties of the peptides were enhanced, and their solubility was improved. Conversely, the emulsification and foaming abilities of the peptides were reduced. The functional activities of the peptides, such as antioxidant and antiglycemic properties, were also enhanced. This study provides a reference for the utilization of high F-ratio peptides in food applications.

## Highlights

• Preparation of high F-ratio peptides using sea buckthorn seed meal protein.
• Decrease in aromatic amino acids, leading to structural changes in peptides.
• The decrease in hydrophobicity affects the physical and chemical properties of peptides.
• High F-ratio peptides have better functional activities than normal peptides.

## 1 Introduction

Seabuckthorn has been identified as a high-quality source of bioactive peptides, owing to its wide range of amino acids and high percentage of essential amino acids (Yushipitsina et al., 1988). Seabuckthorn is a plant that is native to India. It has been found that the total protein content of various species of seabuckthorn ranges from 46 to 129 g/kg dry weight (DW). The protein content of the Polish variety has been documented as 93 g/kg DW. Its protein content is notably higher than that of strawberry (*Fragaria ananassa* Duch.), golden berry (*Solanum mammosum* L.) and raspberry (*Rubus ideaus* L.) seed meals. Seabuckthorn seed meal is a by-product of the supercritical CO_2_ extraction process of seabuckthorn oil. At the moment, most of this by-product is used as animal feed or is thrown away, which is a waste of resources(Zheng et al., 2024). Noteworthily, Plant proteins can be converted into peptides by either enzymatic hydrolysis or chemical treatment. These peptides typically contain 2–50 amino acid residues and have a molecular weight of 50–10,000 Da. Due to their small molecular weight, peptides are more easily digested and absorbed by the human body. A growing body of research is demonstrating the biological activities of plant-derived peptides, including antioxidant, antimicrobial, anti-inflammatory, immunomodulatory, anti-hypertensive and hypoglycemic functions. These findings suggest a potential for use in health and medical applications, such as improving digestion, enhancing immunity, anti-aging and preventing cardio-cerebral vascular diseases.

The molar ratio between branched chain amino acids (BCAA) and aromatic amino acids (AAA) is denoted by the F-ratio, where BCAA includes valine, leucine and isoleucine and AAA includes phenylalanine, tryptophan and tyrosine(Zhang et al., 2022). High F-ratio peptides are defined as small peptides with a value greater than 20 and a peptide number greater than 2 and less than 20 (Ying et al., 2023). Currently, high F-ratio peptides can be obtained from diverse protein sources, including plant proteins such as those found in maize, hempseed, wheat gluten, soybeans and flaxseed; proteins of aquatic origin such as Antarctic krill; and proteins of milk origin such as goat whey and casein hydrolysate. However, the majority of studies on high F-ratio peptides have focused exclusively on their impact on functional activity, neglecting to consider the structural characteristics of these peptides. Furthermore, there is a paucity of research addressing changes in peptide functionality and conformation.

The present study aims to address this issue by focusing on the preparation of high F-ratio peptides from seabuckthorn seed meal proteins. The study will compare the structure, physical and chemical properties, flavour sensory and biofunctional properties of these high F-ratio peptides, with the aim of providing a reference for their application in food products and other products.

## 2 Materials and methods

### 2.1 Materials

The reagents used in the experiments were analytical grade reagents and did not require any treatment other than experimental. Sea buckthorn seed meal protein was provided by Puredia Limited (Xining, Qinghai, China), Amino acid(20AA，BYA9010) and all the reagent kits used in the experiments were purchased from Beijing Solarbio Science & Technology Co., Ltd. (Beijing, China). Enzyme preparations are available from Angel Yeast Co. (Yichang, Hubei, China), The rest of the drugs are procured with Nanjing Jianjian Bioengineering Institute (Nanjing, China).

### 2.2 Preparation of FPs

The seabuckthorn seed meal protein should be weighed at 1 g, dissolved in distilled water according to the specified proportion, and subjected to enzymatic hydrolysis at the optimal pH and temperature of the selected endopeptidase. It is imperative to ensure that the pH and temperature of the solution remain constant during the enzymatic hydrolysis process. Once the protein hydrolysate meets the specified requirements, it must be removed from solution and placed in boiling water for a duration of 15 min. Subsequently, the pH and temperature must be adjusted in accordance with the enzymatic conditions of the exopeptidase. The hydrolysis should then be carried out at a constant temperature. Finally, the water bath should be boiled for 15 min, cooled to room temperature, and then used.

### 2.3 Preparation of HFPs

The protein peptides extracted from seabuckthorn seed powder were subjected to a series of centrifugation and pH adjustment steps. First, the samples were subjected to a 10 min centrifugation process at 4 °C and 5000 rpm. Following this, the resultant upper layer was adjusted to a pH of 3 using a pH meter, and then placed in a water bath at 30 °C. Subsequently, activated carbon (200 mesh) was added at a solid-liquid ratio of 1:25. Following the adsorption process, the precipitate was removed by means of centrifugation at a temperature of 4 °C and at a rotational speed of 8000 rpm for a period of 15 min. Thereafter, the remaining liquid was filtered through a 0.22 μm aqueous filter and subsequently lyophilized.

### 2.4 Amino acid composition and F-ratio determination

The amino acid composition, as well as the F-ratio, were analysed using an automatic amino acid analyzer (L-8900, Hitachi (China) Ltd.) according to the method of Zheng et al. (2023). Prior to analysis, HCl was utilized for the purpose of hydrolyzing the peptides in order to measure the levels of Cys and Met. Meanwhile, NaOH was employed to hydrolyze the peptides for the purpose of measuring Try. The procedure involved the weighing of 50 mg of peptide powder, The samples were then placed in a polytetrafluoroethylene (PTFE) hydrolysis tube, with 20 mL of 4 M NaOH solution or 20 mL of 6 M HCl added. The test tube containing the specimen was subjected to an airless N_2_ for a period of 1 min, with the objective of eliminating O_2_. The samples were then placed in an oven at 110 °C for 24 h. The analysis was conducted using the following methods: peak area quantification: Area, column type: cation-exchange chromatography column (#2622), calculation method: standard extraction method, and detector: UV–visible detector. The F-ratio was calculated based on the molar ratio of BCAA to AAA.

### 2.5 Determination of peptide yield

The trichloroacetic acid nitrogen solubility index (TCA-SNI) method was employed, and slight modification. Insoluble proteins in solution were precipitated with trichloroacetic acid, and following centrifugation, the nitrogen content in the resultant pellet was determined using the Kjeldahl nitrogen method. The specific method entailed the addition of 1 mL of protein hydrolysate to 3 mL of trichloroacetic acid solution, followed by shaking and mixing for a period of 15 min. The mixture was then subjected to centrifugation at a speed of 1043 x*g* for a duration of 10 min. Thereafter, the upper layer was collected, and a Protein Content Assay Kit (Biuret Method) was employed to ascertain the peptide content. The formula for the calculation of polypeptide yield is as follows:

$$\text{Yield of peptides}\;\left(\%\right)=\frac{n_{1}}{n}\times 100\%$$

where, n_1_: peptide content in the supernatant; n: the amount of protein consumed.

### 2.6 Purification by ultrafiltration

Given that high F-ratio peptides characteristically necessitate peptide chains comprising 2–20 amino acid residues, and considering the average relative molecular mass of amino acids is 128, it can be deduced that the relative molecular mass of the target peptide should range from 256 Da to 2560 Da. The crude high F-ratio peptide prepared was separated by ultrafiltration (M20, Alfa Laval (Shanghai) Technology Co.) through an ultrafiltration membrane with a molecular weight cut-off of 3 kDa, in order to obtain components with molecular weights of <3 kDa. The peptide was then concentrated by rotary evaporation in a rotary evaporator, and finally freeze-dried into powder.

### 2.7 Molecular weight distribution determination and gel filtration chromatography

Dextran gels (20 g, Sephadex G-25; Solarbio Technology) were subjected to a swelling process by being immersed in distilled water at 30 °C for a period of 12 h. Thereafter, the gels were meticulously packed into a column (1.6 × 90 cm) and equilibrated using de-distilled water. The gel was meticulously packed into a column (1.6 × 90 cm), taking care to avoid the presence of air bubbles, and equilibrated for a period of 2 h with the use of de-distilled water. The gel was then eluted with 1.0 mL/min distilled water, and the eluate was collected using a 3 mL centrifuge tube. Elution curves were prepared using the absorption curves of OD_220_ and OD_280_, and the components with high F-ratio were selected for the next experiments. The standards utilized for the construction of the molecular weight correction curves encompassed bovine insulin (MW5733), vitamin B_12_ (MW1355), oxidized glutathione (MW612), and phenylalanine (MW165), which were employed as standard molecular weight proteins.

### 2.8 Fourier transform infrared (FTIR) spectrum

According to the method of Lin et al. (2023), with slight modification, the samples with 2 mg were mixed of 200 mg of dry KBr, ground uniformly with an onyx mortar, pressed and scanned (VERTEX 70, Bruker Beijing Technology Co.) with a Fourier infrared spectrometer. The background was used with KBr, and the scanned spectra were in the range of 4000–400 cm^−1^. The subsequent processing of the data was conducted using Origin software, incorporating limit correction and smoothing for normalisation, with the objective of correcting for the non-linear baseline bias, and PeakFit software was used to calculate the secondary structure of the peptide.

### 2.9 Fluorescence chromatography

Fluorescence spectra (RF6000, Shimadzu (China) Ltd.) were measured in accordance with the method outlined in Habinshuti et al. (2021), with minor adjustments. Specifically, a 5 mg/mL solution of the peptide was prepared using deionized water, which was then utilized for fluorescence spectroscopy. The excitation wavelength employed in this process was 347 nm, and the resulting spectra were recorded using a fluorescence spectrophotometer. The spectral range was from 200 nm to 400 nm, with a constant slit width of 5 nm.

### 2.10 Particle size measurement

The present study employed a laser particle size analyzer (LS 13320, Beckman Coulter Trading (China) Co.) to determine the particle size distribution of HFP and FP. Each lyophilised sample was dissolved in phosphate buffer (10 mM, pH 7.0) to a concentration of 0.1 mg/mL, and then analysed at 25 °C.

### 2.11 Scanning electron microscope (SEM)

Scanning electron microscopy images were obtained using a SEM 450 thermal field emission scanning electron microscope (Nano SEM-450, Thermo Fisher Scientific). The peptide powder was fixed to a metal stage using conductive adhesive and covered with gold powder. The samples were then observed at an accelerating voltage of 15 kV.

#### 2.11.1 Volatile compound (VOC) identification

The method of Habinshuti et al. (2021) was employed, with minor modifications. The volatile compounds were analysed by gas chromatography–mass spectrometry (GC–MS) (GCMS-QP2010 Ultra, Shimadzu (China) Ltd.) and placed into 20 mL vials, which were then sealed with screw caps. Following equilibration at 50 °C for 30 min, the volatile compounds were absorbed by fibres via headspace solid phase microextraction (HS-SPME). The desorption of the volatile compounds was then achieved by inserting the fibres into an Agilent GC inlet (Agilent Technologies, Santa Clara, California, USA) in splitless mode at 250 °C, following a separation period of 3 min. The separation process was carried out using a DB-WAX capillary column (60 × 0.25 mm, 0.25 μm; J & W Scientific, Folsom, CA) with a helium carrier gas at a flow rate of 1.8 mL/min and a total ionic current of 40–450 amu. The volatile compounds were identified using the mass spectrometry database of the National Institute of Standards and Technology (NIST11) and expressed as a percentage of relative peak area for each compound.

#### 2.11.2 Electronic nose (*E*-nose) measurement

E-nose analyses were performed using an e-nose machine (PEN 3.5, AIRSENSE) as described by Yu et al. (2018). 2 mL of HFP or FP were placed in airtight vials and sealed with screw caps. A zero-gas generator is then used to rapidly remove the gaseous compound from the headspace of the vial for measurement.

#### 2.11.3 Electronic tongue (*E*-tongue) measurement

Peptides were configured into a 1:25 (*w*/*v*) peptide solution using deionized water. The solution was then subjected to a centrifugal process at 10,000 *g* for a period of 10 min, after which it was filtered through a 45 μm membrane. Analysis was then performed using an E-tongue (SA402B, INSENT Corporation, Japan) with the following sensory attributes: bitter, sour, fresh and salty.

### 2.12 Physico-chemical properties analysis

#### 2.12.1 Solubility

The method of Yang et al. (2023) was employed with minor modifications to configure HFP and FP into a 5 mg/mL solution using deionized water, 30 min standing. Centrifugation at 8000 *g* for 20 min was then conducted. The resultant upper layer was then utilized to ascertain the protein concentration employing a BCA protein assay kit (Beyotime Biotechnology, Shanghai, China). The protein concentration was subsequently measured at 562 nm using an enzyme marker at varying pH levels. The solubility was determined by the following formula.

$$\text{Solubility}\;\left(\%\right)=\frac{A_{1}}{A_{0}}\times 100\%$$

It is imperative to note that A_0_ and A_1_ denote the protein content of the solution before and after solution centrifugation, respectively.

#### 2.12.2 Emulsification activity (EAI) and emulsion stability index (ESI)

The EAI and ESI of FP and HFP were determined according to the method of Pi et al. (2023)., with slight modifications. Specifically, the solution was configured as a 2.5 mg/mL peptide solution using deionized water and the solution was mixed with soybean oil in a ratio of 3:1 (*V*/V) using a magnetic stirrer at 1000 rpm for 20 min, followed by a 10 min resting time and then mixed with SDS solution (0.1 %, *w*/*v*) in a ratio of 4:1. Finally, the mixture's absorbance was measured at 500 nm. EAI and ESI were determined using the following equations:

$$\mathrm{EAI}\left(m^{2}/g\right)=\frac{2\times 2.303\times A_{0}\times D}{N\times C\times 10000}$$

$$\mathrm{ESI}\left(\min\right)=\frac{A_{0}\times 10}{A_{0}-A_{10}}\times 100\%$$

where A_0_ and A_10_ are the absorbance of the emulsion at 0 min and 10 min, respectively; D is the dilution factor; N represents the volume of oil; and C is the protein content (g/mL).

#### 2.12.3 Foaming capacity (FC) and foam stability (FS)

The FC and FS of HP and HFP were determined according to the method of Pi et al. (2023) with slight modifications. The peptides were reconstituted into a 2.5 mg/mL solution using phosphate buffer (10 mM, pH 7.0), and then homogenised using an Ultra Turrax T25 high performance disperser. The volumes of the samples were recorded at 0 min (V_0_) and 10 min (V_10_), respectively. The FC and FS were determined using the following equations:

$$\mathrm{FC}\left(\%\right)=\frac{V_{0}-V}{V}\times 100\%$$

$$\mathrm{FS}\left(\%\right)=\frac{V_{10}-V}{V_{0}-V}\times 100\%$$

### 2.13 Peptide identification

Initially, the samples were subjected to a reduction, alkylation, and desalting process. Subsequent to this, the treated samples were analysed by LC-MS/MS (Q Exactive focus, Thermo Fisher Scientific) to obtain the raw results of mass spectrometry. Thereafter, analysis was conducted by Byonic software to obtain the identification results. The specific steps involved are outlined below: (1) Sample pretreatment: The purified peptide powder was dissolved in ultrapure water, and dithiothreitol solution was added to the appropriate amount of sample to reach a final concentration of 10 mmol/L. The sample was then reduced in a water bath at 56 °C for 1 h. Thereafter, iodoacetamide solution was added to achieve a final concentration of 50 mmol/L, and the sample was then subjected to a 40-min reaction under light protection. The sample was subsequently desalted using a self-filling desalting column, and the solvent was then evaporated in a vacuum centrifuge and concentrator at 45 °C. The solvent was then evaporated in a vacuum centrifuge concentrator at 45 °C. (2) The following conditions were to be observed for capillary liquid chromatography: chromatographic column: pre-column C18 (300 μm × 5 mm), analytical column C18 (150 μm × 150 mm); mobile phase A: 0.1 % formic acid; mobile phase B: 0.1 % formic acid, 80 % ACN; flow rate: The gradient elution procedure was initiated with a flow rate of 600 nL/min. The initial phase (0–2 min) was characterised by a proportion of buffer B of 4–8 %, followed by the intermediate phase (2–45 min) where the proportion increased to 8–28 %. The final phase (45–55 min) involved a return to the initial proportion of 28 % buffer B. The final concentration of the solution was 50 mmol/L. The reaction was exposed to light for 40 min. B; 45–55 min, 28–40 % B; 55–56 min, 40–95 % B; 56–66 min, 95 % B. (3) Mass spectrometry conditions: voltage 2.2 kV, temperature 270 °C; primary mass spectrometry parameters: resolution 70,000, mass scan range 300–1800 *m*/*z*, AGC target: 3e^6^; secondary mass spectrometry parameters: The resolution of 17,500, which pertains to the product ion scan range (>100 m/z), is characterised by an activation type. CID, Minimum Signal Required: 1500, AGC target: 1e^5^, Maximum IT: 60 ms, Top N: 20, NCE/stepped NCE: 27. (4) Database search: using *Hippophae rhamnoides* as the keyword, download the peptide database, use the software Proteome Discoverer 2.1 to identify and select peptides with high confidence.

### 2.14 Prediction of peptide properties

The activity of peptides was predicted using Peptideranker (http://distilldeep.ucd.ie/PeptideRanker/). Peptideranker scores are determined based on an n-1 neural network probabilistic algorithm, and peptides are designated as potentially active when the score exceeds a 0.5 threshold. The NovoPro tool (https://www.novopro.cn/tools/calc_peptide_property.html) predicts the isoelectric point and hydrophobicity of peptides.

### 2.15 In vitro antioxidant activity of peptides

#### 2.15.1 DPPH radical scavenging rate

The DPPH radical scavenging rate of the samples was determined by reference to method of Liu et al. (2025), with slight modifications. A volume of 0.2 mL of the sample solution, with a concentration of 2 mg/mL, was combined with 2 mL of DPPH anhydrous ethanol solution (recorded as A_1_) and 2 mL of anhydrous ethanol (recorded as A_0_), respectively. Ethanol was utilized in place of the samples (recorded as A_2_), and the reaction was conducted at 30 °C in a condition that prevented exposure to light for a duration of 30 min. The measurement of the absorbance was then taken at a wavelength of 517 nm. The formula employed to calculate the scavenging rate of DPPH radicals is as follows:

$$\text{DPPH radical scavenging rate}=1-\frac{A_{1}-A_{0}}{A_{2}}$$

#### 2.15.2 ABTS radical scavenging rate

ABTS radical scavenging was determined according to method of Liu et al. (2025), with minor modifications. A 2.45 mmol/L potassium persulfate solution and a 7 mmol/LABTS solution were prepared, and the two solutions were subsequently mixed and stored at room temperature, in the dark, for 12 h. The ABTS working solution was then prepared by diluting the solution with phosphate buffer, and an absorption value of 0.7 ± 0.02 was obtained at 734 nm. The measurement of the aforementioned value was conducted at 734 nm by combining 2 mL of the ABTS solution with 0.02 mL of a 2 mg/mL sample solution, followed by a reaction period of 6 min in an environment that was protected from light. Deionized water was utilized in lieu of the sample for the control group. The ABTS free radical scavenging rate was calculated as follows:

$$\text{ABTS radical scavenging rate}=\frac{A_{0}-A_{0}}{A_{0}}$$

where A_1_ is the absorbance in presence of sample and A_0_ is the absorbance of control reaction.

#### 2.15.3 Superoxide anion radical scavenging rate

The rate of scavenging of superoxide anions by the samples was determined through the implementation of a Superoxide Anion Scavenging Capacity Assay Kit. The resulting measurement of the sample's absorption was then obtained at 550 nm according to the method stipulated in the kit's instructions.

#### 2.15.4 Iron reducing power

The ferric reducing power of the samples was determined according to the method of Liu et al. (2025), with slight modifications. To this end, 2 mL of 0.2 mol/L phosphate buffer and 2 mL of potassium ferricyanide solution (1 g/100 mL) were added to 0.2 mL of sample solution (4 mg/mL). The mixture was then incubated in a water bath at 50 °C for 20 min. Thereafter, the reaction was stopped with 2.5 mL of TCA (10 *g*/100 mL). Following centrifugation at 6000 r/min for 10 min, the resulting 2.5 mL of upper layer was mixed with 2.5 mL of deionized water and 0.5 mL of ferric chloride (100 mg/100 mL). The mixture's absorbances were measured at 700 nm, and a positive correlation was observed between the absorbances and the iron reducing power.

### 2.16 Statistical analysis

The IBM SPSS Statistics 27 software was utilized for the analysis of all data, with the Tukey multiple comparison test and single factor ANOVA employed to evaluate any discrepancies in values. Furthermore, the PeakFit software was employed to calculate the peptide secondary structure, and the Origin 2018 and GraphPad Prism 10.1 software packages were used to create images. All data calculated three or more times; a statistically significant difference was considered to be *p* < 0.05.

## 3 Results and discussion

### 3.1 Preparation of high F-ratio peptides

In peptides, the amide bond produces an absorption peak at 180–230 nm, where among them amide I (π → π*) absorbs at 190 nm and amide II (n → π*) absorbs at 220 nm. In the near-ultraviolet region, aromatic residues such as tyrosine, tryptophan, phenylalanine, and hemibladder acid have absorption peaks at 240–290 nm. It is evident that the utilization of this property of peptides can be optimised for protein enzymatic assays. The preparation of high F-ratio peptides is primarily concerned with the adsorption of aromatic amino acids. The underlying principle is related to the nature of these amino acids, and intermolecular forces play a pivotal role in the adsorption process. In general, hydrophobic interactions play a dominant role for nonpolar substances, while hydrogen bonding and electrostatic interactions are more important for most ordinary or charged substances, and van der Waals forces exist between almost all substances, but their influence is extremely weak. Aromatic amino acids possess a benzene ring within their molecular structure, enabling them to absorb specific wavelengths of ultraviolet light. This property distinguishes them from other amino acids and has been found to be associated with hydrophobic interactions and π-π interactions between the graphite ring and the benzene ring. These interactions are known to be the predominant factors in the adsorption of AC to the benzene ring. The process involves the reaction of the hydroxyl and carboxyl groups on the AC with the various functional groups present on the benzene ring. This results in the formation of hydrogen bonds and electrostatic interactions. Consequently, the adsorption of aromatic amino acid residues by activated carbon is more efficient than that of other amino acid residues(Pignataro et al., 2020).

In this experiment, the optimised data is shown in Fig. S1a variety of endopeptidases, including alkaline protease, acid protease, neutral protease, and conformational protease, as well as two exopeptidases, i.e. pineapple protease and papain, were screened for proteolytic enzymes. The hydrolysis degree and the OD_220_/OD_280_ ratio were identified as the primary influencing factors. Ultimately, alkaline protease was selected for the endopeptidase assay, and papain for the exopeptidases. The high F-ratio peptides and a single-factor test were used for the enzyme. The final endopeptidase digestion time was 234 min, the substrate concentration was 6.6 %, and the enzyme activity was 5900 U/g, and the exopeptidase digestion time was 164 min, and the enzyme activity was selected as 5132 U/g. The use of activated charcoal was employed for the adsorption of aromatic amino acids from the peptides. The final yield of ordinary peptides was determined to be 85.47 ± 1.34 %, while the yield of peptides following activated charcoal adsorption was 22.54 ± 0.86 %.

### 3.2 Amino acid composition analysis

The amino acid compositions of HFP and FP are presented in the following Table 1. By subjecting seabuckthorn seed meal proteins to enzymatic hydrolysis using alkaline protease and papain, and adsorption using activated charcoal, the content of aromatic amino acids was significantly reduced. The most abundant hydrolyzed amino acids were found to be Glu/Gln, with an increase in BCAA from 12.59 % to 17.25 %. Conversely, the content of AAA exhibited a decline from 6.53 % to 1.17 %, with Trp demonstrating the most substantial decrease of 97.07 %, followed by Phe with a reduction of 90.77 %. The F-ratio was determined to be 20.379, thereby satisfying the criterion for high F-ratio peptides with an F-ratio exceeding 20.

**Table 1:** Amino acid composition table.

Amino acid	Content in FP (g/100 g)	Content in HFP (g/100 g)	Amino acid	Content in FP (g/100 g)	Content in HFP (g/100 g)
Asp/Asn	6.366	4.563	Met	0.177	0.057
Thr	1.534	1.302	Ile	1.984	1.140
Ser	2.638	1.788	Leu	3.569	2.324
Glu/Gln	14.757	8.317	Tyr	1.393	0.154
Pro	1.391	1.069	Phe	1.918	0.177
Gly	2.162	1.085	His	1.148	0.556
Ala	2.072	1.860	Lys	1.468	0.936
Val	2.415	1.774	Arg	8.622	3.245
Trp	0.8197	0.024	Hydrophobic	15.685	10.446
BCAA	7.968	5.238	AAA	4.131	0.355
F-ratio	2.802		20.379		

Note: Hydrolysis of peptides with HCl or hydrolysis of peptides with NaOH will hydrolyze asparagine and glutamine to aspartic acid and glutamic acid.

### 3.3 Molecular weight distribution of peptides

**Fig. 1:**
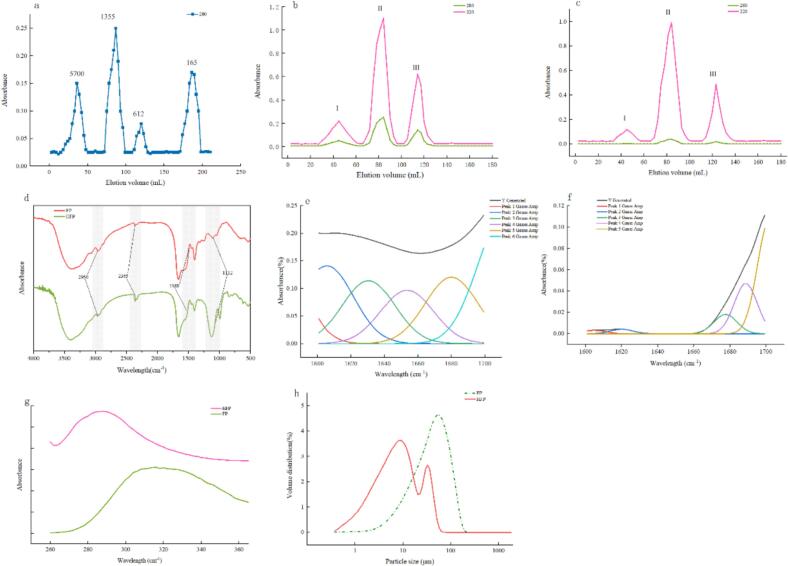
Structural profiles of the peptides. a. molecular weight distribution of the specimen; b. molecular weight distribution of FP; c. molecular weight distribution of HFP; d. fourier infrared chromatograms of the peptides; e. Gaussian fit to the secondary structure of FP; d. Gaussian fit to the secondary structure of HFP; e. Fluorescence chromatograms of the peptides; f. Particle size distributions of the peptides.

The proteolytic solution obtained by ultrafiltration was chromatographed using Sephadex G-25 gel filtration, and the fractions of each tube were collected. The resulting chromatographic curves are shown in Fig. 1 b and Fig. 1 c. It is evident from the figure that there was no significant difference in molecular weight distribution between the two, in which the 1355 Da molecular weight contained the most peptide content. In order to meet the requirements of a high F-ratio peptide, the enzyme digest between component III and group IV was selected for the next step of the study.

### 3.4 FT-IR spectrum

FT-IR spectroscopy can also be used to determine changes in the functional groups of mixed peptides before and after the preparation of high F-ratio peptides. The infrared spectra of peptides show multiple bands generated by peptide bonding in addition to other expected signals. The two most frequently examined regions of the spectrum are the amide I (1,600–11,700 cm^−1^) and amide II (1480–1580 cm^−1^) peaks. The stronger amide I band originates mainly from C=O stretching vibrations, with a smaller contribution from C—N stretching. The weaker amide II band originates from the bending motion of the N—H group and the stretching of the C—N bond of the peptide bond. The amide I and amide II bonds are very sensitive to the content of the secondary structure and its changes due to the direct involvement of the C=O and N—H bonds of the peptide in the main chain conformation and hydrogen bonding pattern of the peptide(Dai et al., 2024). As demonstrated in the infrared spectra of FP and HFP (see Fig. 1 d), no novel functional groups were observed to be produced following adsorption by activated charcoal. However, fluctuations at 2950 cm^−1^ (C—H vibration) were observed. The observed fluctuation is hypothesized to be the consequence of a diminution in the quantity of peptides that occurs during the adsorption process. The alterations in peptide secondary structures were discerned through the implementation of Gaussian fitting analysis at 1600–1700 cm^−1^, which revealed the presence of three secondary structures within the FP: namely, β-folding, β-turning, and α-helix. It was observed that the proportion of β-turning within the HFP increased by 60.14 % relative to the FPs. Concurrently, the number of β-folding structures diminished by 43.92 %, and the α-helix structure became non-existent. This phenomenon may be attributed to the interaction between aromatic amino acids and the peptide secondary structure(Nomoto et al., 2022). The benzene ring structure of aromatic amino acids facilitates the formation of π-π stacking interactions, which contribute to the stabilization of the β-folded structure. Consequently, aromatic amino acids are observed to be present with high frequency in the context of the β-folded structure. Moreover, in certain instances, aromatic amino acids have been observed to stabilise the formed α-helical structure through interactions with other amino acids. For instance, the hydrophobic nature of aromatic amino acids may facilitate the formation of a hydrophobic core within the α-helix, thereby enhancing the stability of the α-helical structure(Nomoto et al., 2022).

### 3.5 Fluorescence spectral analysis

The intrinsic fluorescence of peptides is fundamentally attributable to the aromatic amino acids' phenylalanine, tyrosine and tryptophan. However, due to the fact that tryptophan has a quantum yield one or even two orders of magnitude higher compared to tyrosine and phenylalanine, almost only tryptophan fluorescence is observed in peptides containing all three amino acids simultaneously (Agro, 1974). It is important to note that the maximum wavelength of tryptophan in proteins exhibits significant variation, ranging from 310 to 369 nm (Khrustalev et al., 2019).As demonstrated in Fig. 1 g, the fluorescent spectra of FP and HFP are presented. The shift in maximum absorption, which may be attributed to a ‘blue shift’ phenomenon, could be indicative of a change in solvent phase properties, with HFP exhibiting an optimal solvent phase more acidic than that of FP (Khrustalev et al., 2019), During the activated charcoal adsorption process, the optimal adsorption pH was acidic due to the selection of activated charcoal, which resulted in the precipitation of another spot of peptide under acidic conditions, which was more easily bound to the activated charcoal, so that the prepared HFP had high solubility under acidic conditions. Due to the inherent characteristics of fluorescence, the change in the intensity of fluorescence of aromatic amino acids is found to be in accordance with Beer-Lambert Law (Pignataro et al., 2020). That is to say, the fluorescence intensity displays a positive, albeit non-linear, correlation with the content of aromatic amino acids. It can be deduced that the decrease in the degree of absorption at the uppermost point of HFP relative to that of FP absorption depicted in Fig. 1g is a consequence of the decline in HFP aromatic amino acids.

### 3.6 Particle size analysis

Changes in particle size are closely related to structural changes between peptides or functional changes in protein hydrolysate. The particle size distributions of FP and HFP are illustrated in Fig. 1 h, the average particle size of the HFP is evidently reduced (*p* < 0.05), but compared with the FP, the distribution range of the HFP is more uneven, which is consistent with the phenomenon observed using the electron microscope. Two PSD peaks are evident. This phenomenon may be attributed to a reduction in aromatic amino acids and the peptide segments within the hydrophobic interactions are reduced, resulting in dispersion(Nomoto et al., 2022). However, certain peptides that originally possessed no hydrophobic amino acids remain unchanged, thereby producing two peaks.

### 3.7 Microstructure of peptides

**Fig. 2:**
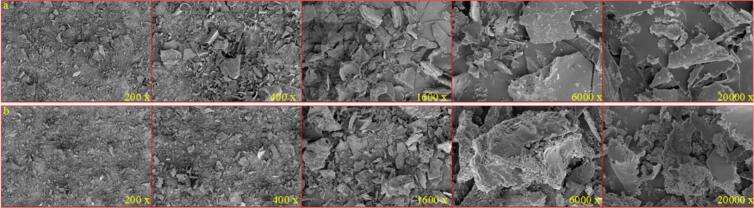
SEM mapping of the peptides. a. Micrograph of FP; b. Micrograph of HFP.

The scanning electron microscope images of the FP and the HFP are shown in Fig. 2. It is evident from the figure that under microscopic conditions, the FP has a tighter structure and stronger agglomeration, while the HFP has a looser structure and weaker agglomeration relative to the FP. At 12,000, the surface of the HFP is more rounded and smoother relative to the FP, enhancing the peptide's contact with the solution, reducing its propensity to clump in the solvent, and facilitating dissolution.

### 3.8 Peptide sensory flavour evaluation

Enzymatic reactions of protein hydrolysates yield short peptides and free amino acids. Alterations in pH, enzymatic reactions, and fluctuations in temperature have been observed to result in the generation of distinctive flavour precursors. The mechanism of the reaction principally includes decarboxylation and deamination of amino acids and peptides, which have formed aldehydes, sulfides, and aromatic compounds. Notably, the Strecker reaction of phenylalanine and leucine has been identified as a predominant source of aldehydes, including hexanal and heptanal(Habinshuti et al., 2021). Arginine, histidine and other basic amino acids have been found to be molecularly correlated with the formation of 2-phenylethanol, ethyl acetate and ethyl benzoate (Yang et al., 2022).

#### 3.8.1 VOCs of peptides

**Fig. 3:**
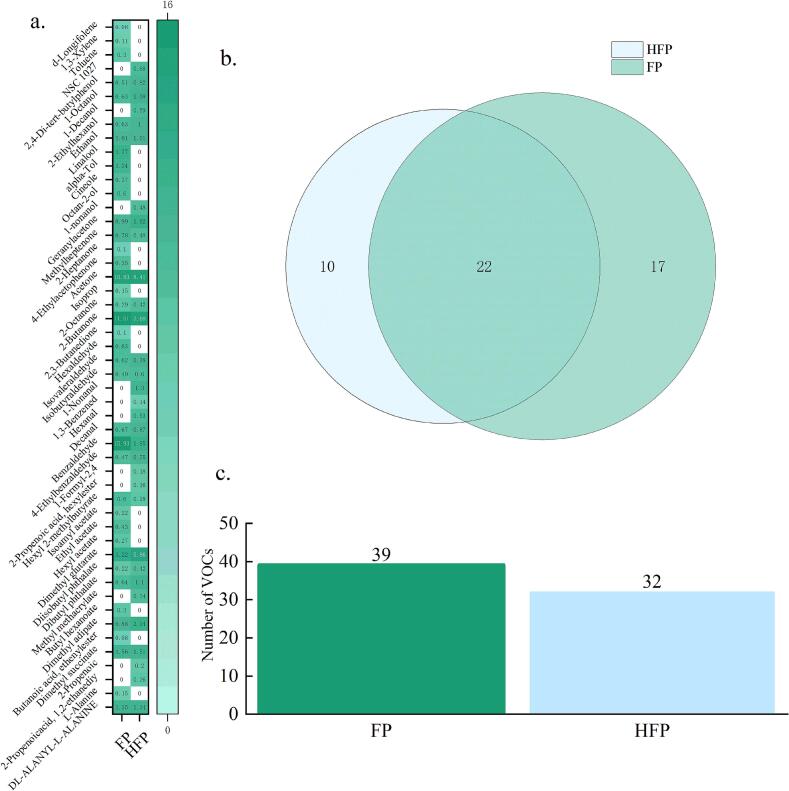
a. Heat map of volatile matter content; b. Wayne diagram of FP vs. HFP volatiles; c. Plot of FP vs. HFP volatiles.

A total of 49 compounds were identified from FPs and HFPs by GC/MS analysis, among which 39 substances were detected in FP, mainly containing 13 esters, 6 aldehydes, 9 ketones, 7 acids, 1 phenol, 2 acids and other alkanes. In addition, 32 substances were identified in HFP, comprising 11 esters, 5 ketones, 9 aldehydes, 5 alcohols, 1 acid, and other substances. The alterations in the substance content of the volatiles are demonstrated in Fig. 3.

Prior to and following the preparation, the content of benzaldehyde underwent the most substantial change, and it has been demonstrated that benzaldehyde is the primary volatile substance for peptide flavour production (Sonklin et al., 2011), with a content of 15.93 % in FP, but decreased to 1.85 % in HFP, exhibiting a significant decline in content (*p* < 0.05), which may be attributable to the reduction in the content of aromatic amino acids in HFP. The most prevalent constituent substances in both substances were esters, which may be related to the enzymatic digestion of proteins using pineapple protease. This has been documented as a catalyst for the esterification of carboxylic acids with alcohols, producing esters under specific conditions. The adsorption process using activated charcoal led to an increase in aldehyde species, which may be attributable to α-keto acids, such as carboxylic acids. These acids have been shown to convert phenylalanine to benzaldehyde through the Strecker reaction. However, the absence of phenylalanine in the HFP suggests that alternative pathways may be responsible for its degradation, leading to the production of the observed aldehydes (Hidalgo et al., 2013).

#### 3.8.2 *E*-nose analysis

**Fig. 4:**
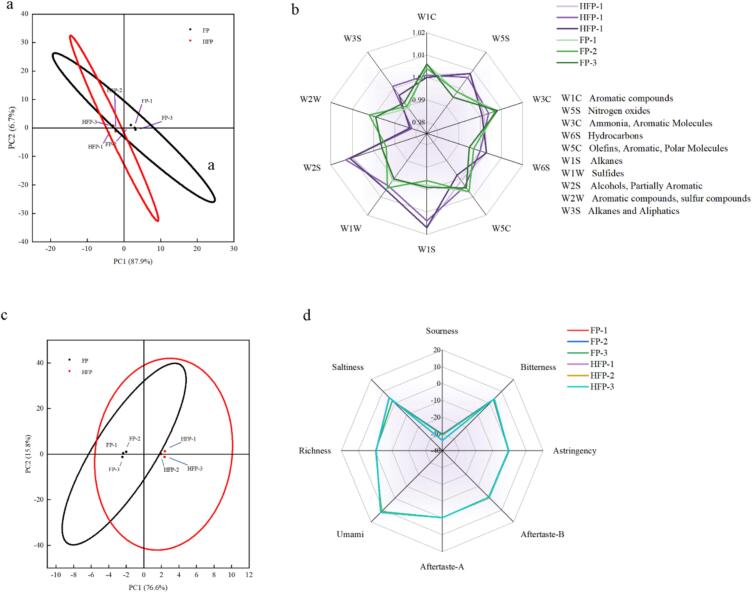
a) Electronic tongue principal component analysis plot; b) electronic nose radar plot; c) electronic tongue principal component analysis plot; d. Electronic tongue radar plot.

The Fig. 4 a with a variance of 94.6 % explains the aroma profile between FP and HFP, with 87.9 % of PC1 components and 6.7 % of PC2 components. These components overlap with each other and are in close proximity to each other, suggesting that there is no significant difference (*p* > 0.05) in odour between the two. The radar chart reveals the presence of our results, indicating the use of GC/MS. The aromatic volatile products in the HFP are reduced, yet this does not affect the difference in odour between the two. It is important to note that the sulphide component in the volatile gases will cause discomfort. The preparation of HFP odour in the sulphide gas is reduced, indicating that the preparation of HFP will reduce some of the discomfort odour production.

#### 3.8.3 E-tongue analysis

The Fig. 4 c with a variance of 92.4 % explained the flavour profile between FP and HFP, which overlapped each other, indicating that there was no obvious significant difference (*p* > 0.05), based on the radar plot between the two, it can be seen that both peptides had smaller data in the e-tongue test, and it is interesting to note that the freshness, which is a sensory feature, was more pronounced as compared to the other sensory organoleptic features, which may be attributed to the fact that both peptides contained peptides with small and medium molecular weights This may be due to the high content of small and medium molecular weight peptides in both peptides (Su et al., 2012). This suggests that the two peptides have the potential to prepare fresh flavour peptides and do not produce an unpleasant taste on the palate when added to foods.

### 3.9 Physico-chemical functional analysis

#### 3.9.1 Solubility

**Fig. 5:**
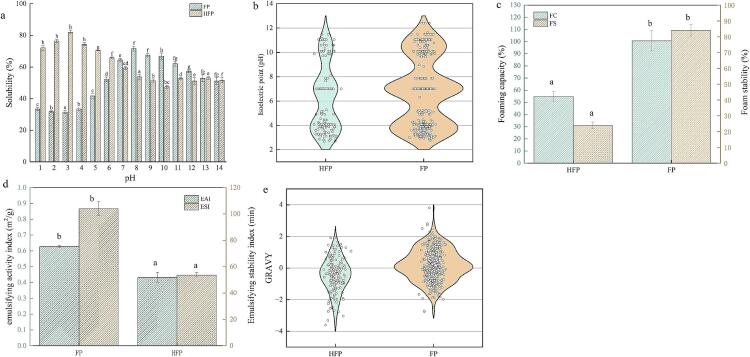
Physicochemical property analysis of peptides. a. Solubility of peptides; b. Isoelectric point distribution of peptides; c. Foaming ability and foam stability of peptides; d. Emulsification and emulsion stability of peptides; e. Hydrophobicity distribution of peptides.

Solubility is a pivotal functional property of bioactive peptides, which provides an index of thermodynamic equilibrium between protein/peptide-protein/peptide and protein/peptide-solvent interactions(Patil et al., 2022). The solubility of peptides is a critical factor in determining their ability to be recognised by intestinal oligopeptide transporters (e.g. PEPT1) as monomers and subsequently transported across membranes. Peptides with a molecular weight of less than 500 Da and which contain charged residues (e.g. glutamate, lysine) are more readily absorbed due to their high solubility, and their bioavailability is more than 50 % higher than that of the aggregated state. In contrast, unsolubilized peptides are prone to forming a β-folding or gel state, which can mask the active site and lead to the failure to penetrate the cell membrane(Patil et al., 2022). The solubility of peptides is induced by the strong interaction of polar groups with water molecules and is essential for many other functional aspects of proteins. The solubility of peptides at varying pH levels is demonstrated in Fig. 5 a. The FPs exhibited enhanced solubility under alkaline conditions, while the HFP demonstrated higher solubility under acidic conditions. This phenomenon can be attributed to the optimal adsorption pH of the activated carbon used, which was 3, and the isoelectric point of FPs being adsorbed under peptides in acidic conditions.

As illustrated in Fig. 5 b, the predicted isoelectric points of the two peptides are demonstrated. The predicted isoelectric points of the resulting peptides indicate that the isoelectric points of the peptides in FP are approximately 4, 7 and 10, while those of the peptides in HFP are predominantly concentrated around 4. This discrepancy is inconsistent with the solubility results that have been obtained, and it has previously been reported in the literature that protein degradation in smaller peptides typically results in higher solubility, particularly during hydrolysis across a range of different pH values. However, it should be noted that not all peptides exhibit significant solubility and solubilisation capacity; this property is contingent upon the total hydrophobicity and charge of the peptide. This is a disadvantageous limitation when considering a hydrolysate comprising hundreds of peptides. The underlying cause of this phenomenon may be attributed to the fact that, while the number of peptides predicted to possess acidic isoelectric points is substantial, their relative abundance is less than that of the peptides with alkaline isoelectric points. This results in peptides with alkaline total isoelectric points. Furthermore, it was observed that the solubility of the HFP was enhanced in comparison to that of the FPs. This phenomenon may be attributed to the reduction of aromatic amino acids, which are hydrophobic amino acids, resulting in a peptide structure that is more hydrophilic than its solubility (Bogahawaththa et al., 2019). The decrease in hydrophobic sites and increase in charge density has been shown to increase the water solubility of peptides (Warnakulasuriya & Nickerson, 2018). Furthermore, significant electrostatic repulsion and hydration of ions may contribute to the increase in peptide solubility at pH values below and above the isoelectric point.

#### 3.9.2 EAI and ESI

EAI is defined as the capacity of a peptide to adsorb oil through the oil-water interface and generate an emulsion (Gorguc et al., 2020). ESI is defined as the ability to stabilise an emulsion over a period of time without coagulation or flocculation. A comparison of the EAI and ESI of the two peptides is shown in Fig. 5 d. It is evident that both EAI and ESI are significantly lower (*p* < 0.05) for HFP compared to FP. This is attributable to the necessity of the hydrophobic and hydrophilic groups within the peptide maintaining equilibrium between the oil and hydrophilic phases to facilitate emulsion formation. However, the significant reduction of aromatic amino acids in HFP disrupts the equilibrium, thereby compromising its capacity to generate an emulsion. Predictions of peptide hydrophobicity, based on Fig. 5 e, indicate that the majority of the hydrophobicity of FP is distributed around 0 and exhibits a symmetrical trend, while the majority of the hydrophobicity of HFP is distributed in negative values. The overall value is shifted towards negative values compared to FP, indicating that a significant number of peptides exhibit hydrophilicity, which is consistent with the observed phenomenon.

#### 3.9.3 FC and FS

The phenomenon of foaminess is contingent upon the hydrophobicity of the gas-liquid interface, the flexibility of the protein molecules, and the solubility and denaturation of the proteins. The three variables of foam formation include migration, penetration and rearrangement of molecules at the gas-water interface. It is imperative that peptides migrate rapidly to the gas-water interface in order to exhibit optimal foaming, unfolding and rearrangement properties. Foam stability is known to correlate with film properties and to reflect the level of protein-protein interactions in the matrix(Karami & Akbari-adergani, 2019). As the Fig. 5 c illustrates, the FC and FS of HFP are significantly different (*p* < 0.05) and weaker than that of FP. This disparity can be attributed to the higher solubility of FP under neutral conditions, which facilitates enhanced peptide participation in the foaming process within the aqueous solution. Additionally, the presence of a greater proportion of hydrophobic amino acids in FP enables expeditious migration of the peptides to the air-water interface, consequently augmenting surface tension in the aqueous solution and enhancing FS.

### 3.10 Evaluation of peptide functional activity

**Fig. 6:**
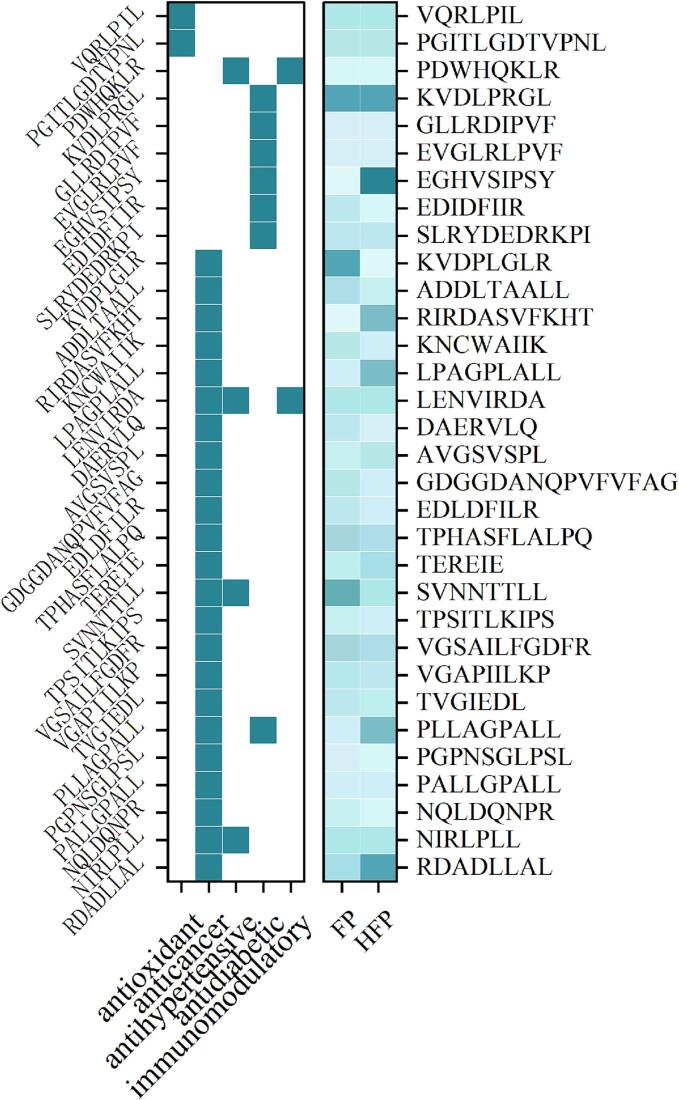
Functional activity distribution map of peptides. Note, the left side represents whether the peptide has the functional activity or not, and the numbers on the right side of the table represent the peptide abundance values.

Bioactive peptides have been demonstrated to exert a variety of effects within the body, with the capacity to regulate physiological processes across diverse metabolic pathways within the organism. Fischer ratio peptides exhibit distinctive functional activities, attributable to their elevated content of BCAA and diminished content of AAA (Wang et al., 2024). For instance, when addressing hepatic encephalopathy and repairing liver damage, it can balance the Fischer ratio in blood after entering the body, and BCAA forms pseudo-neurotransmitters to regulate the body. BCAA in Fischer ratio peptide connects with the tricarboxylic acid cycle through ketogenesis and gluconeogenesis to provide energy for the body. BCAA in high F-ratio peptide can inhibit bile acids to reduce cholesterol level(Wang et al., 2024). In this study, FP and HFP were analysed by HPLC-MS for evaluation, and peptides with peptide activity greater than 0.5. A total of 32 peptides were analysed, and the secondary mass spectra of these 32 peptides with the structures predicted using AlphaFold tools (Google Inc., California, USA) are shown in S2, as illustrated in Fig. 6, the alteration in the functional activity of the peptide in 32 is evident (*p* < 0.05). Among them, 23 peptides had anticancer effects, such as RDADLLAL, NIRLPRLL, NQLDQNPR, PALLGPALL, PGPNSGLPALL, PLLAGPALL, etc. With regard to peptide content, the abundance of four anticancer peptides was increased, among which LPAGPLALL, PLLAGPALL, RDADLLAL contained significant quantities of branched-chain amino acids and did not contain aromatic amino acids. The most extensively studied anticancer peptide is lunasin, which is derived from soybeans and also contains substantial quantities of branched-chain amino acids, while being devoid of aromatic amino acids. The present study guessing that high F-ratio peptides may exert an effect on anticancer, and some studies have demonstrated that contemporary research on the correlation between BCAAs and cancer focuses on enzymes related to catabolism. The precise mechanism of action varies for different malignancies. Branched-chain amino acid transferase enzymes (BCATs) are responsible for the initial step in the catabolism of BCAAs, with two existing BCAT isoforms, cytoplasmic BCAT1 and mitochondrial BCAT2, capable of converting BCAAs to the corresponding branched-chain α-keto acids and ultimately glutamate. BCAT1 has been identified as a significant isoform, with a strong association with cancer proliferation and elevated activity levels in the majority of tumors (Xu et al., 2023).

Furthermore, the content of antioxidant peptide VQRPLPLL, PGITLGDTVPNL, was found to be significantly elevated (*p* < 0.05), with the content of PGITLGDTVPNL increasing by 48 %. 21 %, both peptides exhibited characteristics analogous to the aforementioned examples, containing branched chain amino acids and being devoid of aromatic amino acids. Antioxidant peptides have been demonstrated to effectively inhibit both enzymatic and non-enzymatic peroxidation reactions through a variety of mechanisms, including the scavenging of free radicals, the chelation of metal ions such as Fe^2+^, the reduction of power, and the inhibition of lipid peroxidation (Falcon et al., 2019; Gherasim et al., 2024; Rajapakse et al., 2005). Antioxidant activity is also related to the size and amino acid composition of the peptide. Peptides with high antioxidant activity contain significant quantities of methionine, leucine, histidine, alanine, and valine, the majority of which are hydrophobic (Chen et al., 1996; Garcia et al., 2013; Guo et al., 2009), which is consistent with the phenomenon observed in this study.

Antidiabetic peptides also demonstrated an increasing trend, with KVDLPRGL, EGHVSIPSY, and PLLAGPALL increasing by 36.72 %, 81.77 %, and 36.47 %, respectively. Some studies have demonstrated that metabolism of branched-chain amino acids (BCAAs) in vivo is associated with diabetes, for example Branched-chain amino acids and a variety of catabolic metabolites have been identified as signalling molecules that activate a range of processes, from protein synthesis to the insulin secretion programmes. However, the precise mechanisms by which they act in vivo remain to be fully elucidated (Neinast et al., 2019). In a seminal study, Mosley et al. (2024) demonstrated a causal relationship between BCAAs and type 2 diabetes (T2D), based on a limited number of genetic loci associated with BCAAs.

It is noteworthy that the AlphaFold tools predictions of peptide structures have also revealed a substantial number of cyclic peptides, including AVGSVSPL, GLLRDIPVF, KVDLPRGL, PDWHOKLR, SVNNTTLL, TVGIEDL, PLLAGPALL, PGPNSGLPSL, NIRLPLL. The occurrence of these cyclic peptides may be attributed to the enzymatic degradation of proteins, as evidenced by numerous studies that have demonstrated the presence of cyclic peptides in protein and peptide hydrolysates. This is thought to be due to non-enzymatic head-to-tail cyclisation from linear peptides that are formed during thermal manipulation, peptide storage, as well as in the frozen state (Borthwick & Da Costa, 2017; De Masi et al., 2023; Martian et al., 2025).However, only three of the nine cyclic peptides exhibited an increase in abundance value: AVGSVSPL, GLLRDIPVF, and PLLAGPALL, by 12.49 %, 29.34 %, and 26. 62 %, respectively, and the majority of them demonstrated a decrease in abundance. This phenomenon may be ascribed to the cyclic structure of the cyclic peptides, which facilitates the proximity of the internal nonpolar groups, thereby enhancing their hydrophobicity and facilitating facile absorption of the cyclic peptides by the activated carbon.

As demonstrated above, the utilization of activated carbon adsorption AAA in the preparation of HFP enhances the biofunctional activity of the peptide in comparison with FP. In addition to its efficacy in treating liver injury and counteractant fatigue, this approach also demonstrates the potential for developing high F-ratio peptides with special amino acid sequences that exhibit anticancer, antioxidant, and antidiabetic properties, however these findings require further validation through in vivo and in vitro experimentation.

### 3.11 In vitro antioxidant test

**Fig. 7:**
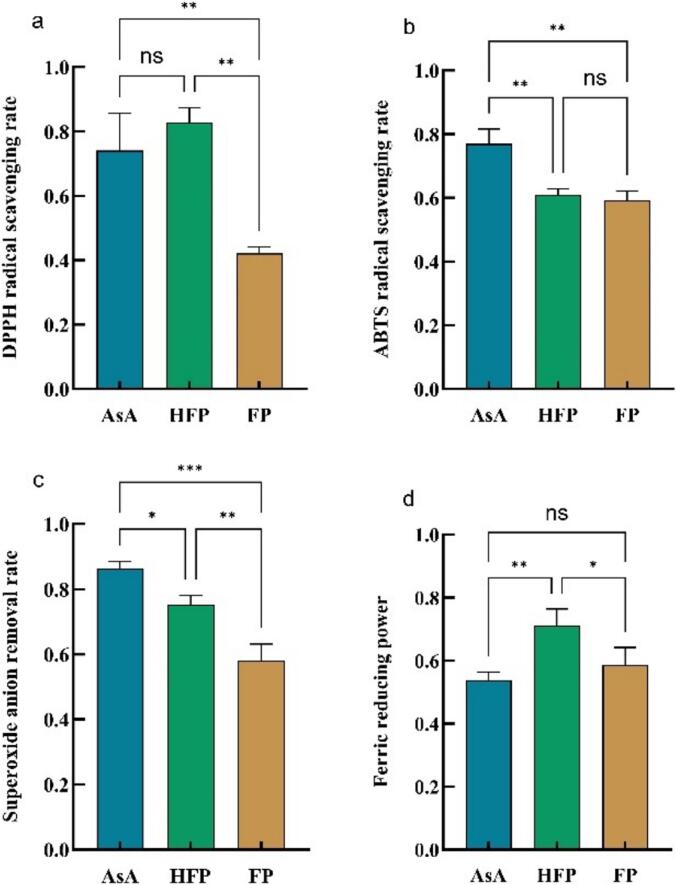
Antioxidant activity of HFP vs. FP. a) DPPH radical scavenging rate, b) ABTS radical scavenging rate, c) Superoxide anion radical scavenging rate, d) Iron reducing power.

In order to verify the predicted functional activities of the peptides, in vitro antioxidant assays were performed on both peptides, the results of which are shown in Fig. 7. The peptides were evaluated for DPPH radical scavenging capacity, ABTS radical scavenging capacity, superoxide anion scavenging capacity, and iron reduction capacity, using ascorbic acid (AsA) as the control group. The results showed that, except for the scavenging rate of the ABTS radicals, both peptides exhibited antioxidant activity that was significantly different from each other. However, a highly significant difference was observed between the DPPH radical scavenging rate and the superoxide anion scavenging rate (*p* < 0.01). The DPPH radical scavenging rate exhibited a 96.23 % increase, the superoxide anion scavenging rate demonstrated a 29.65 % increase, and the iron reduction capacity showed a 21.56 % increase. It is also noteworthy that the iron reduction capacity of HFP was greater than that of the control group, and there was no significant difference between it and the DPPH scavenging rate. In summary, while the computerized prediction of peptide function is feasible, further in vivo and in vitro testing is required for the remaining potential functional activities.

### 3.12 Discussion

The core feature of high F-ratio peptides is that the F-value of BCAA and AAA in their amino acid composition is greater than 20. This special composition results in high F-ratio peptides exhibiting unique functional activities. The present study demonstrates that a reduction in AAA within high F-ratio peptides engenders a series of alterations in their structural, physical, and functional characteristics, with these alterations exerting reciprocal effects on one another.

In the context of peptide secondary structure, the benzene ring structure of aromatic amino acids has been shown to promote the formation of π-π stacking interactions. These interactions have been demonstrated to contribute to the stabilization of the β-folded structure. Consequently, aromatic amino acids have been observed to be present at high frequencies in the context of the β-folded structure. Moreover, in certain instances, aromatic amino acids have been observed to stabilise the formed α-helical structure through interactions with other amino acids. For instance, the hydrophobicity of aromatic amino acids has been shown to promote the formation of a hydrophobic core within the α-helix, thereby enhancing the stability of the α-helix structure (Nomoto et al., 2022).As fluorophores that are intrinsic to peptides, the fluorescence absorption spectra correlate with the content of AAA, and the fluorescence intensity of tryptophan residues reflects the change in hydrophobicity of the peptide, which is due to the adsorption of hydrophobic amino acids during adsorption on activated charcoal. Due to the change in isoelectric point of the peptide during adsorption, which leads to a shift in the wavelength of the maximum absorption of HFP(Nomoto et al., 2022).

It has been demonstrated that the reduction of aromatic amino acids exerts an effect on the peptide particle size and microstructure, among other phenomena. This phenomenon may be attributed to the increased hydrophobicity of the benzene ring side chains in aromatic amino acids (Nomoto et al., 2022). The increased hydrophobicity leads to enhanced aggregation in solution and reduced contact with the surrounding medium, thus promoting stronger agglomerations. In addition, the addition of activated carbon to the peptide solution appears to affect the aggregation behavior of the peptide. This phenomenon can be attributed to the heightened hydrophobicity of peptides containing aromatic amino acid residues, which renders them more susceptible to binding to activated carbon, thereby evading interaction with the aqueous phase. Consequently, peptides with high aggregation propensity in HFP are more likely to be adsorbed by activated carbon, resulting in their loosely. The reduction in particle size may give the peptide a higher biological activity, for instance, Xie et al. (2024) utilized ultrasound-assisted dual enzymatic hydrolysis of camel's milk protein to prepare DPP-IV inhibitory peptides, which exhibited a reduced particle size and enhanced inhibitory activity in comparison to single enzymatic hydrolysis. Similarly, Peng et al. (2024) employed ultrasound treatment of cereal bran protein hydrolysate to demonstrate that decreasing the particle size resulted in enhanced solubility, thermal stability, and antioxidant capacity of the peptides. This finding aligns with the observations presented below, which demonstrate that HFP exhibits greater levels of activity and functionality in comparison with FP.

In terms of sensory analysis, the reduction of the benzene ring led to a decrease in the volatiles of the peptides, particularly phenylalanine, resulting in an increase in aldehydes in HFP. However, no significant differences were observed in odour or taste between the two. In terms of physical and chemical properties, the absence of aromatic amino acids reduced the hydrophobicity of the peptides and had an effect on the solubility, emulsification, and foaming properties of the peptides. This may provide some insight into the fact that when HFP is referred to food products, the best products may be added as ingredients for beverages. In terms of functional activity, both FP and HFP contain a large number of functionally active peptides, which are useful for anticancer, antioxidant, and antidiabetic, etc. However, the peptide function has only been predicted by computer, and the specific principles for these diseases may require us to carry out the next step of the experiment.

In conclusion, the present study advocates for the optimal utilization of seabuckthorn seed meal, thus providing a valuable reference point for the production of high-nutritional products through the use of high F-ratio peptides derived from seabuckthorn seed meal.

## 4 Conclusion

In the present experiment, the synthesis of high F-ratio peptides was conducted for the first time, utilizing seabuckthorn seed meal protein as a substrate. This was accomplished through a single-factor experiment, a response surface optimization test, and activated carbon adsorption. Prior to and following the synthesis of the high F-ratio peptides, a decline in aromatic amino acids was detected, resulting in alterations to the secondary structure, diminished fluorescence chromatogram absorption, and a reduction in volatiles. However, no substantial disparities were identified in the sensory evaluation. Furthermore, the physicochemical properties of the peptides, including solubility, foaming and emulsification, were enhanced. In addition, computer prediction was utilized to estimate the functional activities of the peptides, which showed enhanced anticancer and antidiabetic properties, as well as a significant increase in antioxidant function in vitro, which was subsequently confirmed by experimental validation. However, it must be noted that further in vivo and in vitro tests are required for the validation of other functions.

## CRediT authorship contribution statement

**Yuanju Zheng:** Writing – review & editing, Writing – original draft, Investigation, Data curation. **Yunxi Zhou:** Writing – original draft, Investigation, Data curation. **Yichao Pei:** Writing – review & editing, Data curation. **Zhuoling Feng:** Writing – review & editing. **Michael Yuen:** Writing – review & editing, Funding acquisition, Conceptualization. **Hywel Yuen:** Writing – review & editing, Funding acquisition. **Qiang Peng:** Writing – review & editing, Funding acquisition, Conceptualization.

## Fundings

This research was financially supported by the Science and Technology Project of Xining (Grant No. 2022-Y-12, 2023-Y-05) and Northwest A&F University College Students' Innovation and Entre preneurship Training Programme (Grant No. 202410712287).

## Declaration of competing interest

The authors declare that they have no known competing financial interests or personal relationships that could have appeared to influence the work reported in this paper.

## Data Availability

The authors do not have permission to share data.

## Appendix A Supplementary data

Supplementary material Image 1

## References

[bb0005] Agro A.F. (1974). Intrinsic fluorescence of a protein devoid of tyrosine and tryptophan: Horse hepatocuprein. FEBS Letters.

[bb0010] Bogahawaththa D., Nguyen Hoang Bao C., Trivedi J., Dissanayake M., Vasiljevic T. (2019). Impact of selected process parameters on solubility and heat stability of pea protein isolate [article]. LWT- Food Science and Technology.

[bb0015] Borthwick A.D., Da Costa N.C. (2017). 2,5-diketopiperazines in food and beverages: Taste and bioactivity. Critical Reviews in Food Science and Nutrition.

[bb0020] Chen H.M., Muramoto K., Yamauchi F., Nokihara K. (1996). Antioxidant activity of designed peptides based on the antioxidative peptide isolated from digests of a soybean protein [article]. Journal of Agricultural and Food Chemistry.

[bb0025] Dai S., Xu T., Yuan Y., Fang Q., Lian Z., Tian T., Wang H. (2024). Combination and precipitation mechanism of soy protein and tea polyphenols. Food Hydrocolloids.

[bb0030] De Masi A., Li X.X., Lee D.H.Y., Jeon J., Wang Q., Baek S., Auwerx J. (2023). Cyclo(his-pro): A further step in the management of steatohepatitis. Jhep Reports.

[bb0035] Falcon W.E., Ellingson S.R., Smith J.C., Baudry J. (2019). Ensemble docking in drug discovery: How many protein configurations from molecular dynamics simulations are needed to reproduce known ligand binding? [article]. Journal of Physical Chemistry B.

[bb0040] Garcia M.C., Puchalska P., Esteve C., Marina M.L. (2013). Vegetable foods: A cheap source of proteins and peptides with antihypertensive, antioxidant, and other less occurrence bioactivities [review]. Talanta.

[bb0045] Gherasim C.E., Focşan M., Ciont C., Bunea A., Rugină D., Pintea A. (2024). Stability and bioaccessibility of carotenoids from sea buckthorn pomace encapsulated in alginate hydrogel beads. Nutrients.

[bb0050] Gorguc A., Gencdag E., Yilmaz F.M. (2020). Bioactive peptides derived from plant origin by-products: Biological activities and techno-functional utilizations in food developments - a review [review]. Food Research International.

[bb0055] Guo H., Kouzuma Y., Yonekura M. (2009). Structures and properties of antioxidative peptides derived from royal jelly protein [article]. Food Chemistry.

[bb0060] Habinshuti I., Mu T.-H., Zhang M. (2021). Structural, antioxidant, aroma, and sensory characteristics of Maillard reaction products from sweet potato protein hydrolysates as influenced by different ultrasound-assisted enzymatic treatments. Food Chemistry.

[bb0065] Hidalgo F.J., Delgado R.M., Zamora R. (2013). Intermediate role of α-keto acids in the formation of Strecker aldehydes. Food Chemistry.

[bb0070] Karami Z., Akbari-adergani B. (2019). Bioactive food derived peptides: A review on correlation between structure of bioactive peptides and their functional properties [review]. Journal of Food Science and Technology-Mysore.

[bb0075] Khrustalev V.V., Poboinev V.V., Stojarov A.N., Khrustaleva T.A. (2019). Microenvironment of tryptophan residues in proteins of four structural classes: Applications for fluorescence and circular dichroism spectroscopy. European Biophysics Journal.

[bb0080] Lin Z., Wu H., Zhang M. (2023). Isolation, identification, and structure-activity relationship of novel ACE inhibitory peptides from earthworm protein *in vitro* gastrointestinal digestion product. Food Bioscience.

[bb0085] Liu H., Fan H., Teng X., Sun T., Zhang S., Wang N., Wang D. (2025). Exploring novel antioxidant cyclic peptides in corn protein hydrolysate: Preparation, identification and molecular docking analysis. Food Chemistry.

[bb0090] Martian P.C., Tertis M., Leonte D., Hadade N., Cristea C., Crisan O. (2025). Cyclic peptides: A powerful instrument for advancing biomedical nanotechnologies and drug development. Journal of Pharmaceutical and Biomedical Analysis.

[bb0095] Mosley J.D., Shi M., Agamasu D., Vaitinadin N.S., Murthy V.L., Shah R.V., Ferguson J.F. (2024). Branched-chain amino acids and type 2 diabetes: A bidirectional Mendelian randomization analysis. Obesity (Silver Spring).

[bb0100] Neinast M., Murashige D., Arany Z. (2019). Branched chain amino acids. Annual Review of Physiology.

[bb0105] Nomoto A., Nishinami S., Shiraki K. (2022). Affinity of aromatic amino acid side chains in amino acid solvents. Biophysical Chemistry.

[bb0110] Patil P.J., Usman M., Zhang C., Mehmood A., Zhou M., Teng C., Li X. (2022).

[bb0115] Peng Z., Wang F., Yu L., Jiang B., Cao J., Sun Z., Cheng J. (2024). Effect of ultrasound on the characterization and peptidomics of foxtail millet bran protein hydrolysates. Ultrasonics Sonochemistry.

[bb0120] Pi X., Sun Y., Liu J., Wang X., Hong W., Cheng J., Guo M. (2023). Characterization of the improved functionality in soybean protein-proanthocyanidins conjugates prepared by the alkali treatment. Food Hydrocolloids.

[bb0125] Pignataro M.F., Herrera M.G., Dodero V.I. (2020). Evaluation of peptide/protein self-assembly and aggregation by spectroscopic. Methods.

[bb0130] Rajapakse N., Mendis E., Jung W.K., Je J.Y., Kim S.K. (2005). Purification of a radical scavenging peptide from fermented mussel sauce and its antioxidant properties [article]. Food Research International.

[bb0135] Sonklin C., Laohakunjit N., Kerdchoechuen O. (2011). Physicochemical and flavor characteristics of flavoring agent from Mungbean protein hydrolyzed by bromelain. Journal of Agricultural and Food Chemistry.

[bb0140] Su G., Cui C., Zheng L., Yang B., Ren J., Zhao M. (2012). Isolation and identification of two novel umami and umami-enhancing peptides from peanut hydrolysate by consecutive chromatography and MALDI-TOF/TOF MS. Food Chemistry.

[bb0145] Wang Z., Zhang X., Wang L., Ou X., Huang J. (2024). High Fischer ratio oligopeptides in food: Sources, functions and application prospects. Journal of Future Foods.

[bb0150] Warnakulasuriya S.N., Nickerson M.T. (2018). Review on plant protein-polysaccharide complex coacervation, and the functionality and applicability of formed complexes [review]. Journal of the Science of Food and Agriculture.

[bb0155] Xie Y., Wang J., Wang S., He R., Wang Z., Zhao L., Ge W. (2024). Preparation, characterization, and mechanism of DPP-IV inhibitory peptides derived from Bactrian camel milk. International Journal of Biological Macromolecules.

[bb0160] Xu C., Zhang J., Zhou J., Zheng Y., Huang W., Qin D., Li Y. (2023). Identification, characterization and chemical management of Alternaria alternata causing blackcurrant leaf spot in China. Journal of Applied Microbiology.

[bb0165] Yang X., Wan Q., Wu D., Wang J., Abbas T., Zhang Q. (2023). The impact of novel azotobacter Bacillus sp. T28 combined sea buckthorn pomace on microbial community structure in paddy soil. Environmental Research.

[bb0170] Yang Y.J., Ai L.Z., Mu Z.Y., Liu H.D., Yan X., Ni L., Xia Y.J. (2022). Flavor compounds with high odor activity values (OAV>1) dominate the aroma of aged Chinese rice wine (Huangjiu) by molecular association. Food Chemistry.

[bb0175] Ying Z., Yuyang H., Meiying L., Bingyu S., Linlin L., Mingshou L., Xiuqing Z. (2023). High Fischer ratio peptide of hemp seed: Preparation and anti-fatigue evaluation in vivo and in vitro. Food Research International.

[bb0180] Yu Z., Chen Y., Zhao W., Li J., Liu J., Chen F. (2018). Identification and molecular docking study of novel angiotensin-converting enzyme inhibitory peptides from *Salmo salar* using *in silico* methods [article]. Journal of the Science of Food and Agriculture.

[bb0185] Yushipitsina G.G., Chuprova N.A., Repyakh S.M. (1988). Fractionation and amino acid compositon of proteins of the woody verdure of sea buckthorn. Chemistry of Natural Compounds.

[bb0190] Zhang Z.Y., Qiang F.F., Liu G.Q., Liu C.H., Ai N. (2022). Distribution characteristics of soil microbial communities and their responses to environmental factors in the sea buckthorn forest in the water-wind erosion crisscross region. Frontiers in Microbiology.

[bb0195] Zheng S.-L., Wang Y.-Z., Zhao Y.-Q., Chi C.-F., Zhu W.-Y., Wang B. (2023). High Fischer ratio oligopeptides from hard-shelled mussel: Preparation and hepatoprotective effect against acetaminophen-induced liver injury in mice. Food Bioscience.

[bb0200] Zheng Y., Wang D., Zhou Y., Yuen M., Yuen T., Yuen H., Peng Q. (2024). Characterization and angiotensin-converting enzyme inhibitory activity of peptides of seabuckthorn (Hippophae rhamnoides L.) seed meal. Food Innovation and Advances.

