# Supplementary material for: Preparation of high Fischer ratio peptides from seabuckthorn (Hippophae rhamnoides L.) seed meal, characterization and functional evaluation
Source: Food Chem X. 2025 May 5;27:102523. doi: 10.1016/j.fochx.2025.102523 (PMC12131248; doi:10.1016/j.fochx.2025.102523)
Supplement: Supplementary file 1 — Supplementary material [file mmc1.docx]

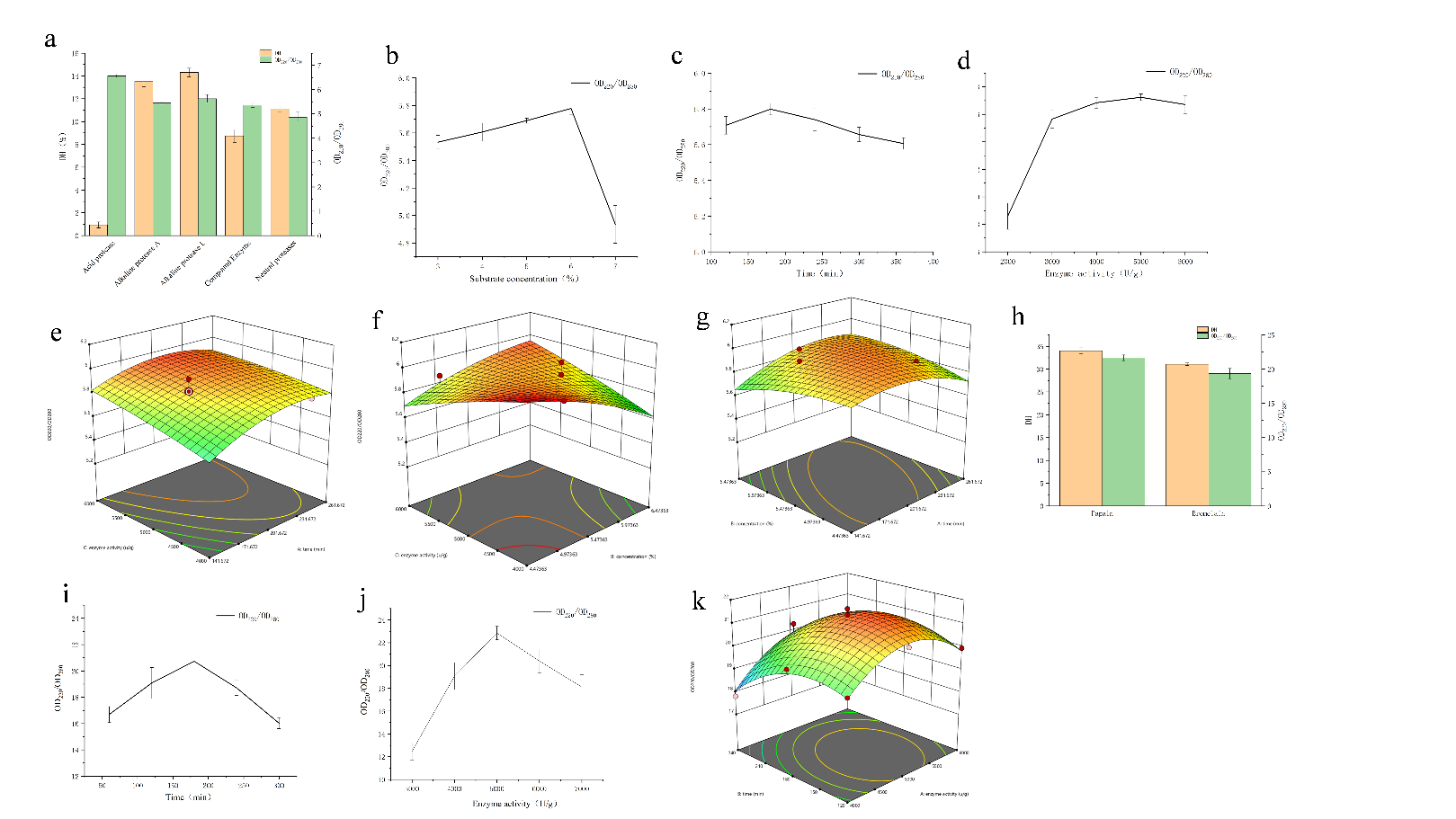


S1. Enzymolysis optimization experiment. a. Endopeptidase single factor test; b. Endopeptidase substrate concentration single factor experiment; c. Endopeptidase enzymolysis time single factor experiment; d. Endopeptidase activity single factor experiment; e-g. Endopeptidase response surface optimization test; h. Exopeptidase single factor test; i. Single factor test of enzymatic hydrolysis time of exopeptidase; j. Exopeptidase activity single factor experiment; k. Exopeptidase response surface optimization test


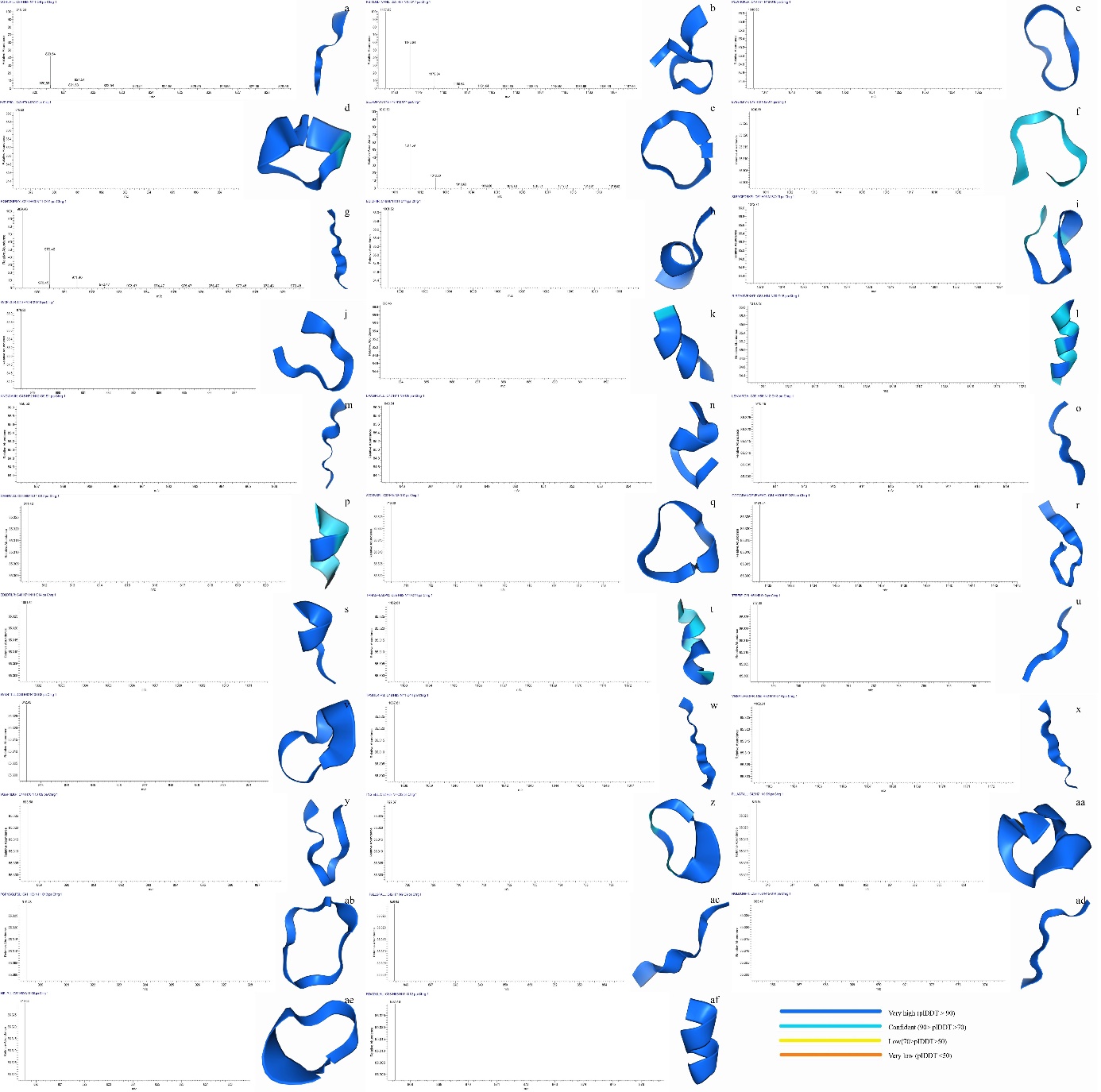


S 2 Secondary mass spectra and 3d structures of 32 identified peptides. pLDDT: a per-atom confidence estimate on a 0-100 scale where a higher value indicates higher confidence.
